# [(4*R*,5*R*)-(2,2-Dimethyl-1,3-dioxolane-4,5-di­yl)bis­(diphenyl­methano­lato)-κ^2^ *O*:*O*′]bis(*N*-methyl­methanamin­ato)titanium(IV)

**DOI:** 10.1107/S1600536812002929

**Published:** 2012-01-26

**Authors:** Leslie Roteta, Joseph M. Tanski

**Affiliations:** a Department of Chemistry, Vassar College, Poughkeepsie, NY 12604, USA

## Abstract

In the title four-coordinate complex, [Ti(C_2_H_6_N)_2_(C_31_H_28_O_4_)], two symmetry-independent mol­ecules are present in the asymmetric unit. The Ti^IV^ atom displays a distorted tetra­hedral geometry, with Ti—O bond lengths ranging from 1.805 (3) to 1.830 (3) Å and O—Ti—O ligand bite angles of 100.16 (12) and 101.36 (12)°. The short Ti—N bond distances, ranging from 1.877 (4) to 1.905 (4) Å, indicate strong bonding between the Ti^IV^ atom and the dimethyl­amide ligands.

## Related literature

For the use of titanium-TADDOLate complexes in asymmetric catalysis, see: Degni *et al.* (2005 ▶); Gothelf *et al.* (1995 ▶); Seebach *et al.* (1992 ▶). For a related structure of a four-coordinate titanium-TADDOLate compound, see: Seebach *et al.* (1992 ▶). For related structures of six-coordinate titanium-TADDOLate compounds, see: Chen *et al.* (2007 ▶); Gothelf *et al.* (1995 ▶); Hinter­mann *et al.* (2002 ▶); Kongprakaiwoot *et al.* (2010 ▶); Shao & Gau (1998 ▶); Shao *et al.* (2001 ▶); Sheen & Gau (2004 ▶). For a report of the *in-situ* preparation of the title compound, see: Ackermann *et al.* (2003 ▶).
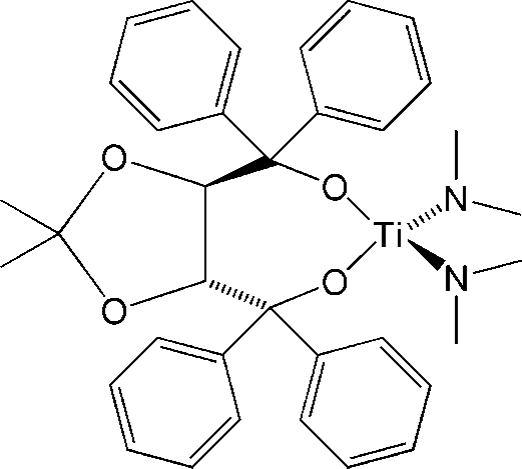


## Experimental

### 

#### Crystal data

[Ti(C_2_H_6_N)_2_(C_31_H_28_O_4_)]
*M* *_r_* = 600.59
Monoclinic, 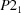

*a* = 9.493 (2) Å
*b* = 21.406 (6) Å
*c* = 15.743 (4) Å
β = 93.562 (4)°
*V* = 3193.0 (14) Å^3^
*Z* = 4
Mo *K*α radiation
μ = 0.31 mm^−1^
*T* = 125 K
0.23 × 0.16 × 0.10 mm

#### Data collection

Bruker APEXII CCD diffractometer
Absorption correction: empirical (using intensity measurements) (*SADABS*; Bruker, 2007 ▶) *T*_min_ = 0.933, *T*_max_ = 0.970
41093 measured reflections
16208 independent reflections
10381 reflections with *I* > 2σ(*I*)
*R*_int_ = 0.073

#### Refinement

*R*[*F* ^2^ > 2σ(*F* ^2^)] = 0.068
*wR*(*F* ^2^) = 0.170
*S* = 1.00
16208 reflections
770 parameters
1 restraint
H-atom parameters constrained
Δρ_max_ = 0.81 e Å^−3^
Δρ_min_ = −0.46 e Å^−3^
Absolute structure: Flack (1983 ▶), with 7788 Friedel pairs; Hooft *et al.* (2008 ▶)
Flack parameter: 0.05 (2); Hooft parameter: 0.055 (15)

### 

Data collection: *APEX2* (Bruker, 2007 ▶); cell refinement: *SAINT* (Bruker, 2007 ▶); data reduction: *SAINT*; program(s) used to solve structure: *SHELXS97* (Sheldrick, 2008 ▶); program(s) used to refine structure: *SHELXL97* (Sheldrick, 2008 ▶); molecular graphics: *SHELXTL* (Sheldrick, 2008 ▶); software used to prepare material for publication: *SHELXTL* and *PLATON* (Spek, 2009 ▶).

## Supplementary Material

Crystal structure: contains datablock(s) I, global. DOI: 10.1107/S1600536812002929/yk2040sup1.cif

Structure factors: contains datablock(s) I. DOI: 10.1107/S1600536812002929/yk2040Isup2.hkl

Additional supplementary materials:  crystallographic information; 3D view; checkCIF report

## Figures and Tables

**Fig. 1.**
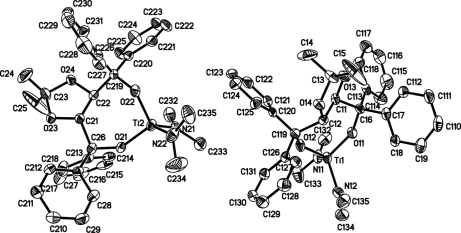
A view of the two independent molecules of the title compound, with displacement ellipsoids shown at the 50% probability level.

## supplementary crystallographic information

### Comment

Titanium(IV) complexes of α,α,α',α'-tetraphenyl-1,3-dioxolane-4,5-dimethanols (TADDOLs) have been reported to be excellent asymmetric catalysts, for example in asymmetric ethylation of aldehydes (Degni *et al.*, 2005; Seebach *et al.*, 1992) and Diels–Alder reactions (Gothelf *et al.*, 1995). Whereas the molecular structure of one four-coordinate Ti(TADDOLate) has been reported (Seebach *et al.*, 1992), six-coordinate complexes are more common (Chen *et al.*, 2007; Gothelf *et al.*, 1995; Hintermann *et al.*, 2002; Kongprakaiwoot *et al.*, 2010; Shao & Gau, 1998; Shao *et al.* 2001; Sheen & Gau, 2004).

The title complex, Ti(TADDOLate)(N(CH_3_)_2_)_2_ is an example of a four-coordinate Ti(TADDOLate) with two dimethylamido ligands. For a report of the *in-situ* preparation of the title compound, see Ackermann *et al.* (2003). The asymmetric unit contains two unique molecules of the title compound (Fig. 1). The complex exhibits a distorted tetrahedral geometry about the titanium(IV) metal center with Ti—O bond lengths ranging from 1.805 to 1.830 Å, average 1.82 (1) Å, Ti—N bond lengths ranging from 1.877 to 1.905 Å, average 1.89 (1) Å, and TADDOLate O—Ti—O bite angles of 100.16 (12)° and 101.36 (12)° for Ti1 and Ti2, respectively. These Ti—O distances are slightly longer than the average Ti—O distance of 1.78 (2) Å found for the 12 Ti—O distances of the three independent molecules in the crystal structure of Ti(TADDOLate)_2_ (Seebach *et al.*, 1992), presumably due to the strongly electron donating dimethylamido groups in the title complex. The observed O—Ti—O bite angles are also slightly less than the average O—Ti—O bite angle of 102.5 (8)° found for the six O—Ti—O bite angles of the three independent molecules in the crystal structure of Ti(TADDOLate)_2_ (Seebach *et al.*, 1992), indicating that titanium in title complex has a more strongly distorted tetrahedral coordination geometry than in Ti(TADDOLate)_2_.

The Ti—O distances in the title complex are also slightly longer than those reported for six-coordinate TADDOLate complexes, where the distances are very similar to those found in Ti(TADDOLate)_2_ (average Ti—O distance 1.78 (2) Å), with TADDOLate Ti—O bond lenghts in six-coordinate complexes 1.780 (2) and 1.786 (1) Å (Chen *et al.*, 2007), 1.76 (1) and 1.79 (1) Å (Gothelf *et al.*, 1995), 1.752 (5) and 1.765 (5) Å (Hintermann *et al.*, 2002), 1.801 (1) and 1.788 (1) Å (Kongprakaiwoot *et al.*, 2010), 1.782 (2) and 1.771 (2) Å (Shao & Gau, 1998), 1.772 (4), 1.778 (3), 1.792 (4) and 1.793 (4) Å (Shao *et al.* 2001), and 1.782 (4), 1.793 (4) and 1.776Å (Sheen & Gau, 2004). However, the observed TADDOLate O—Ti—O bite angles in the title complex, 100.16 (12)° and 101.36 (12)°, are intermediate between those found in Ti(TADDOLate)_2_ (average O—Ti—O bite angle 102.5 (8)°) and those found in six-coordinate complexes, with TADDOLate O—Ti—O bite angles 98.73 (7)° (Chen *et al.*, 2007), 97.2 (5)° (Gothelf *et al.*, 1995), 98.6 (2)° (Hintermann *et al.*, 2002), 94.77 (5)° (Kongprakaiwoot *et al.*, 2010), 98.7 (1)° (Shao & Gau, 1998), 96.8 (2)° (Shao *et al.* 2001), 97.9 (2) and 99.5° (Sheen & Gau, 2004). The six-coordinate complexes presumably exhibit smaller O—Ti—O bite angles because they are distorted octahedral complexes, whereas the title complex is a distorted tetrahedral coordination geometry.

### Experimental

Under a nitrogen atmosphere, tetrakis(dimethylamido)titanium (28.8 mg, 0.13 mmol) was added to a solution of (4*R*,5*R*)-(-)-2,2-dimethyl-α,α,α',α'-tetraphenyl-1,3-dioxolane-4,5-dimethanol (60 mg, 0.13 mmol) in C_6_D_6_ (2.5 ml) and the benzene was allowed to slowly evaporate yielding light yellow plate crystals within 7 d.

### Refinement

All non-hydrogen atoms were refined anisotropically. H atoms on carbon were included in calculated positions and refined using a riding model at C—H = 0.95, 0.98 or 1.00 Å and *U*_iso_(H) = 1.2, 1.5 or 1.2 × *U*_eq_(C) of the aryl, methyl and methine C-atoms, respectively. The extinction parameter (EXTI) refined to zero and was removed from the refinement.

### Crystal data

**Table:**

[Ti(C_2_H_6_N)_2_(C_31_H_28_O_4_)]	*F*(000) = 1272
*M**_r_* = 600.59	*D*_x_ = 1.249 Mg m^−^^3^
Monoclinic, *P*2_1_	Mo *K*α radiation, λ = 0.71073 Å
Hall symbol: P 2yb	Cell parameters from 9992 reflections
*a* = 9.493 (2) Å	θ = 2.3–26.1°
*b* = 21.406 (6) Å	µ = 0.31 mm^−^^1^
*c* = 15.743 (4) Å	*T* = 125 K
β = 93.562 (4)°	Block, colourless
*V* = 3193.0 (14) Å^3^	0.23 × 0.16 × 0.10 mm
*Z* = 4	

### Data collection

**Table:**

Bruker APEXII CCD diffractometer	16208 independent reflections
Radiation source: fine-focus sealed tube	10381 reflections with *I* > 2σ(*I*)
graphite	*R*_int_ = 0.073
φ and ω scans	θ_max_ = 28.7°, θ_min_ = 1.3°
Absorption correction: empirical (using intensity measurements) (*SADABS*; Bruker, 2007)	*h* = −12→12
*T*_min_ = 0.933, *T*_max_ = 0.970	*k* = −28→28
41093 measured reflections	*l* = −21→21

### Refinement

**Table:**

Refinement on *F*^2^	Secondary atom site location: difference Fourier map
Least-squares matrix: full	Hydrogen site location: inferred from neighbouring sites
*R*[*F*^2^ > 2σ(*F*^2^)] = 0.068	H-atom parameters constrained
*wR*(*F*^2^) = 0.170	*w* = 1/[σ^2^(*F*_o_^2^) + (0.0807*P*)^2^] where *P* = (*F*_o_^2^ + 2*F*_c_^2^)/3
*S* = 1.00	(Δ/σ)_max_ < 0.001
16208 reflections	Δρ_max_ = 0.81 e Å^−^^3^
770 parameters	Δρ_min_ = −0.46 e Å^−^^3^
1 restraint	Absolute structure: Flack (1983) and Hooft *et al.* (2008), with 7788 Friedel pairs
Primary atom site location: structure-invariant direct methods	Flack parameter: 0.05 (2)

### Special details

**Table:**

Experimental. A suitable crystal was mounted in a nylon loop with Paratone-N cryoprotectant oil and data set was collected on a Bruker APEXII CCD platform diffractometer. The structure was solved using direct methods and standard difference map techniques, and was refined by full-matrix least-squares procedures on F^2^ with *SHELXTL* Version 6.14 (Sheldrick, 2008).
Geometry. All s.u.'s (except the s.u. in the dihedral angle between two l.s. planes) are estimated using the full covariance matrix. The cell s.u.'s are taken into account individually in the estimation of s.u.'s in distances, angles and torsion angles; correlations between s.u.'s in cell parameters are only used when they are defined by crystal symmetry. An approximate (isotropic) treatment of cell s.u.'s is used for estimating s.u.'s involving l.s. planes.
Refinement. Refinement of *F*^2^ against ALL reflections. The weighted *R*-factor *wR* and goodness of fit *S* are based on *F*^2^, conventional *R*-factors *R* are based on *F*, with *F* set to zero for negative *F*^2^. The threshold expression of *F*^2^ > σ(*F*^2^) is used only for calculating *R*-factors(gt) *etc*. and is not relevant to the choice of reflections for refinement. *R*-factors based on *F*^2^ are statistically about twice as large as those based on *F*, and *R*-factors based on ALL data will be even larger.Hooft *y* 0.055 (15) (*PLATON*) (Hooft *et al.*, 2008)

### Fractional atomic coordinates and isotropic or equivalent isotropic displacement parameters (Å^2^)

**Table:**

	*x*	*y*	*z*	*U*_iso_*/*U*_eq_	
Ti1	0.03272 (8)	0.73012 (3)	0.39591 (4)	0.02688 (17)	
Ti2	0.38094 (8)	0.52132 (3)	0.89491 (4)	0.02711 (17)	
O11	−0.0262 (3)	0.80945 (13)	0.41135 (17)	0.0292 (7)	
O12	0.1033 (3)	0.71166 (12)	0.50377 (17)	0.0310 (7)	
O13	0.0324 (3)	0.89210 (13)	0.61201 (18)	0.0295 (7)	
O14	0.2423 (3)	0.84022 (13)	0.63529 (16)	0.0262 (6)	
O21	0.4530 (3)	0.44266 (13)	0.90383 (17)	0.0276 (6)	
O22	0.3217 (3)	0.53326 (12)	1.00045 (17)	0.0275 (6)	
O23	0.4262 (3)	0.35060 (13)	1.09631 (17)	0.0274 (6)	
O24	0.2176 (3)	0.39760 (12)	1.11985 (17)	0.0276 (6)	
N11	−0.1251 (4)	0.68054 (17)	0.3625 (2)	0.0370 (9)	
N12	0.1737 (4)	0.72338 (18)	0.3154 (2)	0.0369 (9)	
N21	0.2322 (4)	0.52403 (17)	0.8087 (2)	0.0338 (8)	
N22	0.5164 (4)	0.58072 (19)	0.8676 (2)	0.0390 (10)	
C11	0.0221 (4)	0.83876 (18)	0.5574 (2)	0.0235 (8)	
H11A	−0.0439	0.8078	0.5810	0.028*	
C12	0.1731 (4)	0.81117 (18)	0.5625 (2)	0.0235 (8)	
H12I	0.2215	0.8238	0.5105	0.028*	
C13	0.1555 (5)	0.8889 (2)	0.6674 (3)	0.0344 (10)	
C14	0.1102 (7)	0.8682 (4)	0.7549 (3)	0.086 (3)	
H14A	0.0657	0.8270	0.7500	0.129*	
H14B	0.0427	0.8985	0.7755	0.129*	
H14C	0.1932	0.8661	0.7950	0.129*	
C15	0.2326 (6)	0.9494 (3)	0.6646 (7)	0.107 (3)	
H15A	0.2487	0.9600	0.6054	0.161*	
H15B	0.3236	0.9457	0.6973	0.161*	
H15C	0.1764	0.9823	0.6894	0.161*	
C16	−0.0388 (4)	0.86046 (17)	0.4678 (2)	0.0240 (8)	
C17	0.0399 (4)	0.91653 (19)	0.4336 (2)	0.0269 (9)	
C18	0.1361 (5)	0.9090 (2)	0.3703 (3)	0.0313 (10)	
H18A	0.1557	0.8684	0.3497	0.038*	
C19	0.2030 (5)	0.9608 (2)	0.3377 (3)	0.0389 (11)	
H19A	0.2675	0.9551	0.2946	0.047*	
C21	0.4242 (4)	0.40544 (18)	1.0450 (2)	0.0234 (8)	
H21I	0.4922	0.4366	1.0714	0.028*	
C22	0.2711 (4)	0.43052 (18)	1.0504 (2)	0.0231 (8)	
H22A	0.2144	0.4190	0.9971	0.028*	
C23	0.3074 (5)	0.3474 (2)	1.1458 (3)	0.0401 (11)	
C24	0.3554 (9)	0.3561 (5)	1.2371 (4)	0.143 (5)	
H24A	0.4258	0.3896	1.2421	0.215*	
H24B	0.3974	0.3172	1.2595	0.215*	
H24C	0.2745	0.3673	1.2698	0.215*	
C25	0.2331 (7)	0.2875 (3)	1.1261 (8)	0.143 (5)	
H25A	0.2139	0.2838	1.0644	0.214*	
H25B	0.1439	0.2867	1.1543	0.214*	
H25C	0.2926	0.2526	1.1467	0.214*	
C26	0.4700 (4)	0.38821 (18)	0.9542 (2)	0.0231 (8)	
C27	0.6289 (4)	0.36983 (18)	0.9555 (2)	0.0239 (8)	
C28	0.6961 (5)	0.3762 (2)	0.8809 (3)	0.0353 (10)	
H28A	0.6456	0.3928	0.8319	0.042*	
C29	0.8363 (5)	0.3588 (3)	0.8759 (3)	0.0449 (13)	
H29A	0.8817	0.3649	0.8244	0.054*	
C110	0.1773 (5)	1.0198 (2)	0.3669 (3)	0.0397 (11)	
H11D	0.2237	1.0548	0.3441	0.048*	
C111	0.0843 (5)	1.0282 (2)	0.4291 (3)	0.0409 (11)	
H11C	0.0666	1.0691	0.4494	0.049*	
C112	0.0156 (5)	0.9772 (2)	0.4627 (3)	0.0343 (10)	
H11B	−0.0486	0.9836	0.5058	0.041*	
C113	−0.1980 (5)	0.87705 (19)	0.4680 (3)	0.0299 (9)	
C114	−0.2743 (5)	0.8783 (2)	0.3894 (3)	0.0443 (12)	
H11E	−0.2298	0.8680	0.3389	0.053*	
C115	−0.4176 (6)	0.8950 (3)	0.3857 (4)	0.0528 (14)	
H11F	−0.4705	0.8960	0.3325	0.063*	
C116	−0.4813 (5)	0.9100 (2)	0.4588 (4)	0.0464 (13)	
H11G	−0.5785	0.9210	0.4556	0.056*	
C117	−0.4078 (5)	0.9094 (2)	0.5360 (3)	0.0418 (12)	
H11H	−0.4531	0.9201	0.5861	0.050*	
C118	−0.2631 (5)	0.8926 (2)	0.5407 (3)	0.0330 (10)	
H11I	−0.2110	0.8922	0.5942	0.040*	
C119	0.1837 (4)	0.73799 (19)	0.5734 (2)	0.0254 (9)	
C120	0.1253 (4)	0.71638 (17)	0.6568 (2)	0.0227 (8)	
C121	−0.0140 (5)	0.69568 (18)	0.6580 (3)	0.0297 (9)	
H12H	−0.0723	0.6950	0.6067	0.036*	
C122	−0.0681 (5)	0.6761 (2)	0.7331 (3)	0.0379 (11)	
H12G	−0.1633	0.6623	0.7329	0.046*	
C123	0.0142 (6)	0.6763 (2)	0.8080 (3)	0.0394 (11)	
H12F	−0.0233	0.6624	0.8593	0.047*	
C124	0.1530 (5)	0.6972 (2)	0.8079 (3)	0.0372 (11)	
H12E	0.2108	0.6978	0.8594	0.045*	
C125	0.2059 (5)	0.7168 (2)	0.7334 (3)	0.0340 (10)	
H12D	0.3007	0.7311	0.7342	0.041*	
C126	0.3376 (4)	0.71433 (19)	0.5698 (2)	0.0265 (9)	
C127	0.4564 (5)	0.7523 (2)	0.5767 (3)	0.0328 (10)	
H12C	0.4467	0.7962	0.5827	0.039*	
C128	0.5900 (5)	0.7255 (2)	0.5748 (3)	0.0385 (10)	
H12B	0.6711	0.7515	0.5803	0.046*	
C129	0.6068 (5)	0.6620 (2)	0.5652 (3)	0.0421 (12)	
H12A	0.6985	0.6445	0.5636	0.051*	
C130	0.4887 (5)	0.6240 (2)	0.5577 (3)	0.0364 (11)	
H13B	0.4991	0.5801	0.5516	0.044*	
C131	0.3549 (5)	0.6502 (2)	0.5593 (3)	0.0310 (10)	
H13A	0.2740	0.6241	0.5532	0.037*	
C132	−0.2745 (6)	0.6955 (3)	0.3578 (3)	0.0473 (13)	
H13L	−0.3144	0.6884	0.2997	0.071*	
H13M	−0.2872	0.7394	0.3732	0.071*	
H13N	−0.3229	0.6688	0.3973	0.071*	
C133	−0.0973 (7)	0.6144 (2)	0.3526 (4)	0.0603 (17)	
H13I	−0.1257	0.6016	0.2942	0.090*	
H13J	−0.1512	0.5905	0.3926	0.090*	
H13K	0.0037	0.6064	0.3641	0.090*	
C134	0.1640 (6)	0.6911 (3)	0.2332 (3)	0.0514 (14)	
H13F	0.2432	0.6619	0.2304	0.077*	
H13G	0.1673	0.7218	0.1872	0.077*	
H13H	0.0749	0.6679	0.2270	0.077*	
C135	0.3141 (6)	0.7496 (2)	0.3322 (3)	0.0473 (13)	
H13C	0.3347	0.7787	0.2865	0.071*	
H13D	0.3840	0.7159	0.3349	0.071*	
H13E	0.3182	0.7720	0.3866	0.071*	
C210	0.9099 (5)	0.3328 (2)	0.9456 (3)	0.0412 (12)	
H21A	1.0054	0.3203	0.9421	0.049*	
C211	0.8433 (5)	0.3253 (2)	1.0205 (3)	0.0372 (11)	
H21B	0.8931	0.3073	1.0687	0.045*	
C212	0.7043 (4)	0.3439 (2)	1.0256 (3)	0.0296 (9)	
H21C	0.6598	0.3389	1.0775	0.036*	
C213	0.3831 (4)	0.33496 (19)	0.9133 (2)	0.0272 (9)	
C214	0.2736 (5)	0.3467 (2)	0.8535 (3)	0.0325 (10)	
H21H	0.2524	0.3885	0.8366	0.039*	
C215	0.1942 (5)	0.2973 (3)	0.8180 (3)	0.0429 (12)	
H21G	0.1190	0.3059	0.7770	0.052*	
C216	0.2218 (5)	0.2375 (3)	0.8406 (3)	0.0481 (14)	
H21F	0.1653	0.2046	0.8164	0.058*	
C217	0.3314 (6)	0.2242 (3)	0.8985 (3)	0.0512 (13)	
H21E	0.3523	0.1821	0.9137	0.061*	
C218	0.4123 (6)	0.2729 (2)	0.9352 (3)	0.0423 (12)	
H21D	0.4881	0.2637	0.9755	0.051*	
C219	0.2545 (4)	0.50256 (18)	1.0665 (2)	0.0235 (8)	
C220	0.0979 (4)	0.5229 (2)	1.0584 (2)	0.0242 (8)	
C221	0.0717 (5)	0.5875 (2)	1.0506 (3)	0.0311 (10)	
H22E	0.1485	0.6160	1.0522	0.037*	
C222	−0.0656 (5)	0.6097 (2)	1.0405 (3)	0.0391 (11)	
H22F	−0.0821	0.6533	1.0350	0.047*	
C223	−0.1791 (5)	0.5686 (2)	1.0385 (3)	0.0438 (12)	
H22G	−0.2732	0.5837	1.0307	0.053*	
C224	−0.1524 (5)	0.5050 (2)	1.0480 (3)	0.0437 (12)	
H22H	−0.2295	0.4767	1.0474	0.052*	
C225	−0.0151 (5)	0.4820 (2)	1.0584 (3)	0.0333 (10)	
H22I	0.0009	0.4384	1.0655	0.040*	
C226	0.3224 (4)	0.52279 (19)	1.1527 (2)	0.0266 (8)	
C227	0.4575 (5)	0.5485 (2)	1.1589 (3)	0.0337 (10)	
H22D	0.5086	0.5535	1.1093	0.040*	
C228	0.5170 (6)	0.5668 (2)	1.2375 (4)	0.0535 (15)	
H22C	0.6084	0.5850	1.2410	0.064*	
C229	0.4472 (6)	0.5591 (2)	1.3103 (3)	0.0499 (14)	
H22B	0.4907	0.5713	1.3637	0.060*	
C230	0.3138 (6)	0.5336 (2)	1.3059 (3)	0.0462 (13)	
H23B	0.2651	0.5280	1.3562	0.055*	
C231	0.2505 (5)	0.5160 (2)	1.2269 (3)	0.0384 (10)	
H23A	0.1577	0.4992	1.2237	0.046*	
C232	0.0911 (5)	0.4988 (2)	0.8208 (3)	0.0430 (12)	
H23J	0.0211	0.5324	0.8138	0.064*	
H23M	0.0888	0.4811	0.8781	0.064*	
H23N	0.0690	0.4661	0.7784	0.064*	
C233	0.2411 (6)	0.5473 (2)	0.7222 (3)	0.0492 (13)	
H23K	0.1668	0.5784	0.7099	0.074*	
H23L	0.2290	0.5125	0.6820	0.074*	
H23I	0.3336	0.5667	0.7166	0.074*	
C234	0.6250 (9)	0.5683 (3)	0.8112 (5)	0.092 (3)	
H23C	0.7167	0.5675	0.8435	0.138*	
H23D	0.6252	0.6011	0.7678	0.138*	
H23E	0.6078	0.5277	0.7836	0.138*	
C235	0.5179 (6)	0.6447 (2)	0.8964 (4)	0.0544 (15)	
H23F	0.6123	0.6552	0.9210	0.082*	
H23G	0.4489	0.6499	0.9396	0.082*	
H23H	0.4936	0.6724	0.8482	0.082*	

### Atomic displacement parameters (Å^2^)

**Table:**

	*U*^11^	*U*^22^	*U*^33^	*U*^12^	*U*^13^	*U*^23^
Ti1	0.0365 (4)	0.0189 (4)	0.0242 (4)	−0.0023 (3)	−0.0064 (3)	−0.0005 (3)
Ti2	0.0341 (4)	0.0226 (4)	0.0248 (4)	−0.0068 (3)	0.0028 (3)	0.0041 (3)
O11	0.0395 (17)	0.0232 (15)	0.0239 (15)	−0.0005 (12)	−0.0057 (13)	−0.0022 (11)
O12	0.0474 (18)	0.0175 (14)	0.0269 (15)	−0.0030 (12)	−0.0067 (13)	−0.0006 (11)
O13	0.0317 (16)	0.0258 (15)	0.0299 (15)	0.0038 (13)	−0.0056 (12)	−0.0058 (12)
O14	0.0296 (15)	0.0233 (14)	0.0247 (14)	0.0031 (12)	−0.0081 (12)	−0.0072 (11)
O21	0.0329 (16)	0.0255 (15)	0.0250 (15)	−0.0004 (12)	0.0073 (12)	0.0059 (11)
O22	0.0326 (16)	0.0170 (14)	0.0330 (15)	−0.0065 (11)	0.0027 (12)	0.0043 (11)
O23	0.0303 (15)	0.0250 (15)	0.0275 (15)	0.0020 (12)	0.0076 (12)	0.0078 (12)
O24	0.0294 (15)	0.0221 (14)	0.0319 (16)	0.0034 (12)	0.0078 (12)	0.0057 (12)
N11	0.052 (3)	0.028 (2)	0.030 (2)	−0.0075 (18)	−0.0105 (17)	0.0029 (16)
N12	0.045 (2)	0.029 (2)	0.037 (2)	0.0064 (18)	0.0015 (17)	−0.0029 (17)
N21	0.044 (2)	0.0257 (18)	0.0310 (18)	0.0036 (18)	−0.0014 (15)	0.0036 (16)
N22	0.047 (2)	0.037 (2)	0.034 (2)	−0.0142 (19)	0.0062 (18)	0.0051 (17)
C11	0.029 (2)	0.0183 (19)	0.0226 (19)	−0.0009 (16)	−0.0064 (16)	0.0005 (15)
C12	0.025 (2)	0.024 (2)	0.021 (2)	0.0008 (16)	0.0008 (16)	0.0007 (15)
C13	0.031 (2)	0.027 (2)	0.043 (3)	0.0094 (19)	−0.012 (2)	−0.0128 (19)
C14	0.094 (5)	0.141 (7)	0.024 (3)	0.080 (5)	0.002 (3)	−0.005 (3)
C15	0.039 (3)	0.024 (3)	0.254 (11)	0.000 (3)	−0.023 (5)	−0.037 (4)
C16	0.031 (2)	0.0170 (19)	0.0237 (19)	0.0013 (16)	−0.0001 (16)	−0.0027 (15)
C17	0.032 (2)	0.027 (2)	0.020 (2)	−0.0012 (17)	−0.0117 (17)	0.0037 (16)
C18	0.041 (3)	0.027 (2)	0.025 (2)	0.0042 (19)	0.0000 (19)	0.0052 (17)
C19	0.038 (3)	0.041 (3)	0.037 (3)	−0.008 (2)	−0.006 (2)	0.009 (2)
C21	0.024 (2)	0.023 (2)	0.023 (2)	0.0005 (16)	0.0037 (16)	0.0034 (16)
C22	0.024 (2)	0.026 (2)	0.0190 (19)	−0.0010 (16)	0.0022 (15)	0.0012 (15)
C23	0.036 (3)	0.035 (3)	0.051 (3)	0.014 (2)	0.018 (2)	0.018 (2)
C24	0.134 (7)	0.268 (13)	0.030 (3)	0.151 (8)	0.028 (4)	0.047 (5)
C25	0.058 (4)	0.022 (3)	0.360 (15)	0.006 (3)	0.100 (7)	0.029 (5)
C26	0.026 (2)	0.0183 (19)	0.025 (2)	−0.0098 (16)	0.0020 (16)	0.0020 (15)
C27	0.028 (2)	0.021 (2)	0.023 (2)	−0.0038 (16)	0.0066 (16)	−0.0047 (15)
C28	0.041 (3)	0.041 (3)	0.024 (2)	0.000 (2)	0.0031 (19)	−0.0002 (19)
C29	0.040 (3)	0.061 (4)	0.035 (3)	0.008 (3)	0.013 (2)	0.004 (2)
C110	0.049 (3)	0.028 (2)	0.041 (3)	−0.012 (2)	−0.004 (2)	0.011 (2)
C111	0.064 (3)	0.022 (2)	0.036 (2)	−0.004 (2)	−0.002 (2)	0.0011 (19)
C112	0.049 (3)	0.026 (2)	0.029 (2)	0.001 (2)	0.006 (2)	0.0017 (18)
C113	0.036 (2)	0.018 (2)	0.035 (2)	−0.0008 (17)	−0.0099 (19)	0.0006 (17)
C114	0.040 (3)	0.048 (3)	0.044 (3)	0.013 (2)	−0.011 (2)	−0.005 (2)
C115	0.046 (3)	0.053 (3)	0.057 (3)	0.004 (3)	−0.021 (3)	0.004 (3)
C116	0.036 (3)	0.038 (3)	0.063 (4)	0.000 (2)	−0.011 (3)	0.002 (2)
C117	0.032 (3)	0.039 (3)	0.054 (3)	0.006 (2)	0.002 (2)	0.000 (2)
C118	0.030 (2)	0.037 (3)	0.032 (2)	0.0018 (19)	−0.0007 (18)	0.0013 (19)
C119	0.029 (2)	0.027 (2)	0.0199 (19)	0.0030 (17)	−0.0025 (16)	−0.0021 (16)
C120	0.032 (2)	0.0137 (19)	0.0225 (19)	0.0028 (15)	0.0011 (16)	0.0001 (14)
C121	0.035 (2)	0.021 (2)	0.033 (2)	0.0022 (18)	0.0023 (18)	0.0041 (17)
C122	0.044 (3)	0.019 (2)	0.052 (3)	−0.0006 (19)	0.013 (2)	0.001 (2)
C123	0.060 (3)	0.024 (2)	0.037 (3)	0.003 (2)	0.026 (2)	0.0010 (19)
C124	0.045 (3)	0.040 (3)	0.027 (2)	0.005 (2)	0.003 (2)	0.0003 (19)
C125	0.042 (3)	0.036 (3)	0.023 (2)	−0.002 (2)	−0.0024 (18)	−0.0013 (18)
C126	0.033 (2)	0.028 (2)	0.0186 (19)	0.0033 (17)	−0.0005 (16)	0.0045 (15)
C127	0.035 (3)	0.028 (2)	0.035 (2)	0.0047 (19)	0.0019 (19)	−0.0001 (18)
C128	0.030 (2)	0.043 (3)	0.043 (3)	0.003 (2)	0.0002 (19)	0.000 (2)
C129	0.038 (3)	0.050 (3)	0.039 (3)	0.013 (2)	0.006 (2)	0.006 (2)
C130	0.045 (3)	0.030 (2)	0.035 (3)	0.012 (2)	0.008 (2)	0.0064 (19)
C131	0.035 (2)	0.030 (2)	0.027 (2)	0.0022 (19)	0.0007 (18)	0.0027 (18)
C132	0.055 (3)	0.051 (3)	0.036 (3)	−0.023 (3)	0.003 (2)	−0.008 (2)
C133	0.073 (4)	0.028 (3)	0.076 (4)	−0.011 (3)	−0.031 (3)	0.004 (3)
C134	0.070 (4)	0.054 (3)	0.031 (3)	0.016 (3)	0.001 (2)	−0.006 (2)
C135	0.051 (3)	0.049 (3)	0.042 (3)	−0.009 (2)	0.003 (2)	0.000 (2)
C210	0.039 (3)	0.046 (3)	0.040 (3)	0.008 (2)	0.012 (2)	−0.001 (2)
C211	0.035 (3)	0.041 (3)	0.035 (2)	0.007 (2)	0.001 (2)	0.001 (2)
C212	0.031 (2)	0.033 (2)	0.026 (2)	−0.0002 (19)	0.0087 (17)	0.0029 (18)
C213	0.033 (2)	0.027 (2)	0.022 (2)	−0.0078 (18)	0.0073 (17)	−0.0029 (16)
C214	0.034 (2)	0.038 (2)	0.026 (2)	−0.002 (2)	0.0040 (18)	−0.0101 (18)
C215	0.036 (3)	0.063 (4)	0.030 (2)	−0.013 (2)	0.009 (2)	−0.021 (2)
C216	0.056 (3)	0.053 (3)	0.036 (3)	−0.038 (3)	0.013 (2)	−0.014 (2)
C217	0.080 (4)	0.035 (3)	0.037 (3)	−0.025 (3)	−0.002 (3)	−0.001 (2)
C218	0.063 (3)	0.027 (2)	0.036 (3)	−0.013 (2)	−0.007 (2)	0.0044 (19)
C219	0.027 (2)	0.022 (2)	0.0220 (19)	0.0039 (16)	0.0028 (16)	0.0047 (15)
C220	0.027 (2)	0.025 (2)	0.0201 (18)	0.0018 (18)	−0.0002 (15)	0.0001 (17)
C221	0.038 (3)	0.026 (2)	0.030 (2)	−0.0013 (19)	0.0047 (19)	−0.0020 (17)
C222	0.042 (3)	0.025 (2)	0.051 (3)	0.009 (2)	0.007 (2)	−0.001 (2)
C223	0.036 (3)	0.036 (3)	0.060 (3)	0.010 (2)	0.008 (2)	0.001 (2)
C224	0.028 (2)	0.032 (3)	0.071 (4)	−0.0043 (19)	−0.001 (2)	−0.002 (2)
C225	0.031 (2)	0.022 (2)	0.047 (3)	−0.0017 (17)	0.002 (2)	−0.0007 (19)
C226	0.032 (2)	0.0192 (19)	0.028 (2)	−0.0004 (19)	−0.0041 (16)	0.0030 (18)
C227	0.030 (2)	0.029 (2)	0.041 (3)	−0.0007 (19)	−0.005 (2)	−0.0057 (19)
C228	0.051 (3)	0.041 (3)	0.066 (4)	−0.001 (2)	−0.021 (3)	−0.013 (3)
C229	0.070 (4)	0.038 (3)	0.039 (3)	0.009 (3)	−0.024 (3)	−0.009 (2)
C230	0.075 (4)	0.036 (3)	0.027 (2)	0.009 (3)	−0.003 (2)	−0.0009 (19)
C231	0.054 (3)	0.036 (3)	0.025 (2)	−0.003 (2)	0.0028 (19)	−0.0032 (19)
C232	0.043 (3)	0.039 (3)	0.044 (3)	−0.002 (2)	−0.011 (2)	0.002 (2)
C233	0.071 (4)	0.044 (3)	0.032 (3)	0.011 (3)	−0.002 (2)	0.000 (2)
C234	0.130 (7)	0.062 (4)	0.094 (5)	−0.029 (4)	0.076 (5)	−0.009 (4)
C235	0.052 (3)	0.034 (3)	0.077 (4)	−0.006 (2)	−0.004 (3)	0.010 (3)

### Geometric parameters (Å, °)

**Table:**

Ti1—O11	1.809 (3)	C119—C126	1.551 (6)
Ti1—O12	1.830 (3)	C120—C125	1.387 (6)
Ti1—N11	1.884 (4)	C120—C121	1.396 (6)
Ti1—N12	1.905 (4)	C121—C122	1.384 (6)
Ti2—O22	1.805 (3)	C121—H12H	0.9500
Ti2—O21	1.819 (3)	C122—C123	1.373 (7)
Ti2—N22	1.877 (4)	C122—H12G	0.9500
Ti2—N21	1.897 (4)	C123—C124	1.391 (7)
O11—C16	1.418 (5)	C123—H12F	0.9500
O12—C119	1.414 (5)	C124—C125	1.371 (6)
O13—C13	1.415 (5)	C124—H12E	0.9500
O13—C11	1.429 (5)	C125—H12D	0.9500
O14—C12	1.428 (4)	C126—C127	1.388 (6)
O14—C13	1.439 (5)	C126—C131	1.392 (6)
O21—C26	1.413 (5)	C127—C128	1.394 (6)
O22—C219	1.416 (5)	C127—H12C	0.9500
O23—C23	1.412 (5)	C128—C129	1.378 (7)
O23—C21	1.424 (5)	C128—H12B	0.9500
O24—C23	1.415 (5)	C129—C130	1.384 (7)
O24—C22	1.421 (4)	C129—H12A	0.9500
N11—C133	1.451 (6)	C130—C131	1.391 (6)
N11—C132	1.451 (7)	C130—H13B	0.9500
N12—C135	1.456 (6)	C131—H13A	0.9500
N12—C134	1.465 (6)	C132—H13L	0.9800
N21—C233	1.458 (6)	C132—H13M	0.9800
N21—C232	1.467 (6)	C132—H13N	0.9800
N22—C234	1.426 (7)	C133—H13I	0.9800
N22—C235	1.442 (7)	C133—H13J	0.9800
C11—C12	1.547 (6)	C133—H13K	0.9800
C11—C16	1.561 (5)	C134—H13F	0.9800
C11—H11A	1.0000	C134—H13G	0.9800
C12—C119	1.578 (6)	C134—H13H	0.9800
C12—H12I	1.0000	C135—H13C	0.9800
C13—C15	1.490 (7)	C135—H13D	0.9800
C13—C14	1.533 (8)	C135—H13E	0.9800
C14—H14A	0.9800	C210—C211	1.381 (6)
C14—H14B	0.9800	C210—H21A	0.9500
C14—H14C	0.9800	C211—C212	1.386 (6)
C15—H15A	0.9800	C211—H21B	0.9500
C15—H15B	0.9800	C212—H21C	0.9500
C15—H15C	0.9800	C213—C214	1.381 (6)
C16—C17	1.530 (6)	C213—C218	1.395 (6)
C16—C113	1.552 (6)	C214—C215	1.397 (6)
C17—C112	1.400 (6)	C214—H21H	0.9500
C17—C18	1.402 (6)	C215—C216	1.350 (8)
C18—C19	1.391 (6)	C215—H21G	0.9500
C18—H18A	0.9500	C216—C217	1.369 (8)
C19—C110	1.373 (7)	C216—H21F	0.9500
C19—H19A	0.9500	C217—C218	1.399 (6)
C21—C22	1.557 (5)	C217—H21E	0.9500
C21—C26	1.565 (5)	C218—H21D	0.9500
C21—H21I	1.0000	C219—C226	1.528 (5)
C22—C219	1.572 (5)	C219—C220	1.546 (5)
C22—H22A	1.0000	C220—C225	1.384 (6)
C23—C25	1.487 (9)	C220—C221	1.410 (6)
C23—C24	1.493 (8)	C221—C222	1.387 (6)
C24—H24A	0.9800	C221—H22E	0.9500
C24—H24B	0.9800	C222—C223	1.390 (7)
C24—H24C	0.9800	C222—H22F	0.9500
C25—H25A	0.9800	C223—C224	1.391 (7)
C25—H25B	0.9800	C223—H22G	0.9500
C25—H25C	0.9800	C224—C225	1.394 (6)
C26—C213	1.526 (5)	C224—H22H	0.9500
C26—C27	1.557 (6)	C225—H22I	0.9500
C27—C28	1.379 (6)	C226—C227	1.394 (6)
C27—C212	1.393 (6)	C226—C231	1.397 (6)
C28—C29	1.389 (7)	C227—C228	1.385 (7)
C28—H28A	0.9500	C227—H22D	0.9500
C29—C210	1.380 (7)	C228—C229	1.369 (8)
C29—H29A	0.9500	C228—H22C	0.9500
C110—C111	1.369 (7)	C229—C230	1.377 (8)
C110—H11D	0.9500	C229—H22B	0.9500
C111—C112	1.394 (6)	C230—C231	1.399 (6)
C111—H11C	0.9500	C230—H23B	0.9500
C112—H11B	0.9500	C231—H23A	0.9500
C113—C118	1.376 (6)	C232—H23J	0.9800
C113—C114	1.394 (6)	C232—H23M	0.9800
C114—C115	1.405 (7)	C232—H23N	0.9800
C114—H11E	0.9500	C233—H23K	0.9800
C115—C116	1.370 (8)	C233—H23L	0.9800
C115—H11F	0.9500	C233—H23I	0.9800
C116—C117	1.364 (7)	C234—H23C	0.9800
C116—H11G	0.9500	C234—H23D	0.9800
C117—C118	1.417 (6)	C234—H23E	0.9800
C117—H11H	0.9500	C235—H23F	0.9800
C118—H11I	0.9500	C235—H23G	0.9800
C119—C120	1.529 (5)	C235—H23H	0.9800
			
O11—Ti1—O12	100.16 (12)	C126—C119—C12	112.0 (3)
O11—Ti1—N11	108.59 (16)	C125—C120—C121	117.6 (4)
O12—Ti1—N11	112.09 (15)	C125—C120—C119	122.3 (4)
O11—Ti1—N12	113.36 (15)	C121—C120—C119	120.1 (3)
O12—Ti1—N12	111.68 (16)	C122—C121—C120	120.6 (4)
N11—Ti1—N12	110.56 (17)	C122—C121—H12H	119.7
O22—Ti2—O21	101.36 (12)	C120—C121—H12H	119.7
O22—Ti2—N22	111.83 (15)	C123—C122—C121	120.8 (4)
O21—Ti2—N22	112.64 (17)	C123—C122—H12G	119.6
O22—Ti2—N21	113.16 (15)	C121—C122—H12G	119.6
O21—Ti2—N21	110.05 (15)	C122—C123—C124	119.3 (4)
N22—Ti2—N21	107.80 (17)	C122—C123—H12F	120.4
C16—O11—Ti1	147.8 (2)	C124—C123—H12F	120.4
C119—O12—Ti1	141.5 (2)	C125—C124—C123	119.7 (4)
C13—O13—C11	110.8 (3)	C125—C124—H12E	120.1
C12—O14—C13	110.6 (3)	C123—C124—H12E	120.1
C26—O21—Ti2	146.7 (2)	C124—C125—C120	122.0 (4)
C219—O22—Ti2	142.3 (2)	C124—C125—H12D	119.0
C23—O23—C21	111.7 (3)	C120—C125—H12D	119.0
C23—O24—C22	111.1 (3)	C127—C126—C131	119.0 (4)
C133—N11—C132	113.2 (4)	C127—C126—C119	124.6 (4)
C133—N11—Ti1	115.7 (3)	C131—C126—C119	116.4 (4)
C132—N11—Ti1	130.4 (3)	C126—C127—C128	119.5 (4)
C135—N12—C134	110.5 (4)	C126—C127—H12C	120.2
C135—N12—Ti1	121.5 (3)	C128—C127—H12C	120.2
C134—N12—Ti1	128.0 (3)	C129—C128—C127	121.3 (5)
C233—N21—C232	110.7 (4)	C129—C128—H12B	119.3
C233—N21—Ti2	126.5 (3)	C127—C128—H12B	119.3
C232—N21—Ti2	122.8 (3)	C128—C129—C130	119.4 (4)
C234—N22—C235	112.3 (4)	C128—C129—H12A	120.3
C234—N22—Ti2	123.3 (4)	C130—C129—H12A	120.3
C235—N22—Ti2	124.3 (4)	C129—C130—C131	119.8 (4)
O13—C11—C12	104.2 (3)	C129—C130—H13B	120.1
O13—C11—C16	108.3 (3)	C131—C130—H13B	120.1
C12—C11—C16	116.8 (3)	C130—C131—C126	121.0 (4)
O13—C11—H11A	109.1	C130—C131—H13A	119.5
C12—C11—H11A	109.1	C126—C131—H13A	119.5
C16—C11—H11A	109.1	N11—C132—H13L	109.5
O14—C12—C11	104.7 (3)	N11—C132—H13M	109.5
O14—C12—C119	108.8 (3)	H13L—C132—H13M	109.5
C11—C12—C119	115.9 (3)	N11—C132—H13N	109.5
O14—C12—H12I	109.1	H13L—C132—H13N	109.5
C11—C12—H12I	109.1	H13M—C132—H13N	109.5
C119—C12—H12I	109.1	N11—C133—H13I	109.5
O13—C13—O14	106.7 (3)	N11—C133—H13J	109.5
O13—C13—C15	109.2 (5)	H13I—C133—H13J	109.5
O14—C13—C15	109.0 (4)	N11—C133—H13K	109.5
O13—C13—C14	107.5 (4)	H13I—C133—H13K	109.5
O14—C13—C14	107.8 (4)	H13J—C133—H13K	109.5
C15—C13—C14	116.3 (6)	N12—C134—H13F	109.5
C13—C14—H14A	109.5	N12—C134—H13G	109.5
C13—C14—H14B	109.5	H13F—C134—H13G	109.5
H14A—C14—H14B	109.5	N12—C134—H13H	109.5
C13—C14—H14C	109.5	H13F—C134—H13H	109.5
H14A—C14—H14C	109.5	H13G—C134—H13H	109.5
H14B—C14—H14C	109.5	N12—C135—H13C	109.5
C13—C15—H15A	109.5	N12—C135—H13D	109.5
C13—C15—H15B	109.5	H13C—C135—H13D	109.5
H15A—C15—H15B	109.5	N12—C135—H13E	109.5
C13—C15—H15C	109.5	H13C—C135—H13E	109.5
H15A—C15—H15C	109.5	H13D—C135—H13E	109.5
H15B—C15—H15C	109.5	C29—C210—C211	119.3 (4)
O11—C16—C17	108.7 (3)	C29—C210—H21A	120.4
O11—C16—C113	107.3 (3)	C211—C210—H21A	120.4
C17—C16—C113	108.5 (3)	C210—C211—C212	120.3 (4)
O11—C16—C11	107.2 (3)	C210—C211—H21B	119.9
C17—C16—C11	113.0 (3)	C212—C211—H21B	119.9
C113—C16—C11	111.9 (3)	C211—C212—C27	120.9 (4)
C112—C17—C18	117.8 (4)	C211—C212—H21C	119.6
C112—C17—C16	121.2 (4)	C27—C212—H21C	119.6
C18—C17—C16	121.0 (4)	C214—C213—C218	118.1 (4)
C19—C18—C17	120.2 (4)	C214—C213—C26	121.0 (4)
C19—C18—H18A	119.9	C218—C213—C26	120.9 (4)
C17—C18—H18A	119.9	C213—C214—C215	119.9 (5)
C110—C19—C18	121.0 (5)	C213—C214—H21H	120.0
C110—C19—H19A	119.5	C215—C214—H21H	120.0
C18—C19—H19A	119.5	C216—C215—C214	121.5 (5)
O23—C21—C22	103.4 (3)	C216—C215—H21G	119.3
O23—C21—C26	109.2 (3)	C214—C215—H21G	119.3
C22—C21—C26	116.4 (3)	C215—C216—C217	120.0 (4)
O23—C21—H21I	109.2	C215—C216—H21F	120.0
C22—C21—H21I	109.2	C217—C216—H21F	120.0
C26—C21—H21I	109.2	C216—C217—C218	119.7 (5)
O24—C22—C21	104.5 (3)	C216—C217—H21E	120.2
O24—C22—C219	108.6 (3)	C218—C217—H21E	120.2
C21—C22—C219	116.7 (3)	C213—C218—C217	120.8 (5)
O24—C22—H22A	108.9	C213—C218—H21D	119.6
C21—C22—H22A	108.9	C217—C218—H21D	119.6
C219—C22—H22A	108.9	O22—C219—C226	109.7 (3)
O23—C23—O24	107.0 (3)	O22—C219—C220	106.5 (3)
O23—C23—C25	108.1 (5)	C226—C219—C220	110.1 (3)
O24—C23—C25	109.0 (5)	O22—C219—C22	106.4 (3)
O23—C23—C24	108.6 (5)	C226—C219—C22	112.3 (3)
O24—C23—C24	109.0 (5)	C220—C219—C22	111.6 (3)
C25—C23—C24	114.9 (7)	C225—C220—C221	119.2 (4)
C23—C24—H24A	109.5	C225—C220—C219	124.3 (4)
C23—C24—H24B	109.5	C221—C220—C219	116.5 (4)
H24A—C24—H24B	109.5	C222—C221—C220	120.4 (4)
C23—C24—H24C	109.5	C222—C221—H22E	119.8
H24A—C24—H24C	109.5	C220—C221—H22E	119.8
H24B—C24—H24C	109.5	C221—C222—C223	120.5 (4)
C23—C25—H25A	109.5	C221—C222—H22F	119.7
C23—C25—H25B	109.5	C223—C222—H22F	119.7
H25A—C25—H25B	109.5	C222—C223—C224	118.7 (4)
C23—C25—H25C	109.5	C222—C223—H22G	120.6
H25A—C25—H25C	109.5	C224—C223—H22G	120.6
H25B—C25—H25C	109.5	C223—C224—C225	121.4 (4)
O21—C26—C213	109.8 (3)	C223—C224—H22H	119.3
O21—C26—C27	107.0 (3)	C225—C224—H22H	119.3
C213—C26—C27	108.3 (3)	C220—C225—C224	119.7 (4)
O21—C26—C21	106.8 (3)	C220—C225—H22I	120.1
C213—C26—C21	112.8 (3)	C224—C225—H22I	120.1
C27—C26—C21	111.8 (3)	C227—C226—C231	118.6 (4)
C28—C27—C212	118.2 (4)	C227—C226—C219	120.6 (4)
C28—C27—C26	117.6 (4)	C231—C226—C219	120.8 (4)
C212—C27—C26	124.1 (3)	C228—C227—C226	119.7 (5)
C27—C28—C29	121.2 (4)	C228—C227—H22D	120.1
C27—C28—H28A	119.4	C226—C227—H22D	120.1
C29—C28—H28A	119.4	C229—C228—C227	121.5 (5)
C210—C29—C28	120.2 (4)	C229—C228—H22C	119.2
C210—C29—H29A	119.9	C227—C228—H22C	119.2
C28—C29—H29A	119.9	C228—C229—C230	119.8 (5)
C111—C110—C19	119.7 (4)	C228—C229—H22B	120.1
C111—C110—H11D	120.1	C230—C229—H22B	120.1
C19—C110—H11D	120.1	C229—C230—C231	119.6 (5)
C110—C111—C112	120.4 (4)	C229—C230—H23B	120.2
C110—C111—H11C	119.8	C231—C230—H23B	120.2
C112—C111—H11C	119.8	C226—C231—C230	120.7 (5)
C111—C112—C17	120.8 (4)	C226—C231—H23A	119.7
C111—C112—H11B	119.6	C230—C231—H23A	119.7
C17—C112—H11B	119.6	N21—C232—H23J	109.5
C118—C113—C114	119.9 (4)	N21—C232—H23M	109.5
C118—C113—C16	123.0 (4)	H23J—C232—H23M	109.5
C114—C113—C16	117.1 (4)	N21—C232—H23N	109.5
C113—C114—C115	119.4 (5)	H23J—C232—H23N	109.5
C113—C114—H11E	120.3	H23M—C232—H23N	109.5
C115—C114—H11E	120.3	N21—C233—H23K	109.5
C116—C115—C114	120.1 (5)	N21—C233—H23L	109.5
C116—C115—H11F	120.0	H23K—C233—H23L	109.5
C114—C115—H11F	120.0	N21—C233—H23I	109.5
C117—C116—C115	121.2 (5)	H23K—C233—H23I	109.5
C117—C116—H11G	119.4	H23L—C233—H23I	109.5
C115—C116—H11G	119.4	N22—C234—H23C	109.5
C116—C117—C118	119.3 (5)	N22—C234—H23D	109.5
C116—C117—H11H	120.4	H23C—C234—H23D	109.5
C118—C117—H11H	120.4	N22—C234—H23E	109.5
C113—C118—C117	120.1 (4)	H23C—C234—H23E	109.5
C113—C118—H11I	119.9	H23D—C234—H23E	109.5
C117—C118—H11I	119.9	N22—C235—H23F	109.5
O12—C119—C120	109.8 (3)	N22—C235—H23G	109.5
O12—C119—C126	107.8 (3)	H23F—C235—H23G	109.5
C120—C119—C126	109.0 (3)	N22—C235—H23H	109.5
O12—C119—C12	106.5 (3)	H23F—C235—H23H	109.5
C120—C119—C12	111.7 (3)	H23G—C235—H23H	109.5
			
O12—Ti1—O11—C16	1.0 (5)	C17—C16—C113—C118	−103.4 (4)
N11—Ti1—O11—C16	118.6 (5)	C11—C16—C113—C118	21.9 (5)
N12—Ti1—O11—C16	−118.1 (5)	O11—C16—C113—C114	−43.5 (5)
O11—Ti1—O12—C119	−41.3 (4)	C17—C16—C113—C114	73.9 (5)
N11—Ti1—O12—C119	−156.3 (4)	C11—C16—C113—C114	−160.8 (4)
N12—Ti1—O12—C119	79.0 (4)	C118—C113—C114—C115	−0.6 (7)
O22—Ti2—O21—C26	7.9 (5)	C16—C113—C114—C115	−178.0 (4)
N22—Ti2—O21—C26	127.6 (5)	C113—C114—C115—C116	0.1 (8)
N21—Ti2—O21—C26	−112.1 (5)	C114—C115—C116—C117	0.4 (8)
O21—Ti2—O22—C219	−40.2 (4)	C115—C116—C117—C118	−0.4 (8)
N22—Ti2—O22—C219	−160.5 (4)	C114—C113—C118—C117	0.6 (7)
N21—Ti2—O22—C219	77.6 (4)	C16—C113—C118—C117	177.8 (4)
O11—Ti1—N11—C133	−176.3 (4)	C116—C117—C118—C113	−0.1 (7)
O12—Ti1—N11—C133	−66.6 (4)	Ti1—O12—C119—C120	142.8 (3)
N12—Ti1—N11—C133	58.7 (4)	Ti1—O12—C119—C126	−98.6 (4)
O11—Ti1—N11—C132	−7.3 (4)	Ti1—O12—C119—C12	21.7 (6)
O12—Ti1—N11—C132	102.4 (4)	O14—C12—C119—O12	174.9 (3)
N12—Ti1—N11—C132	−132.2 (4)	C11—C12—C119—O12	57.3 (4)
O11—Ti1—N12—C135	66.9 (4)	O14—C12—C119—C120	55.1 (4)
O12—Ti1—N12—C135	−45.3 (4)	C11—C12—C119—C120	−62.5 (4)
N11—Ti1—N12—C135	−170.9 (3)	O14—C12—C119—C126	−67.5 (4)
O11—Ti1—N12—C134	−115.8 (4)	C11—C12—C119—C126	174.9 (3)
O12—Ti1—N12—C134	132.0 (4)	O12—C119—C120—C125	158.1 (4)
N11—Ti1—N12—C134	6.4 (4)	C126—C119—C120—C125	40.2 (5)
O22—Ti2—N21—C233	146.6 (4)	C12—C119—C120—C125	−84.1 (4)
O21—Ti2—N21—C233	−100.8 (4)	O12—C119—C120—C121	−22.4 (5)
N22—Ti2—N21—C233	22.4 (4)	C126—C119—C120—C121	−140.3 (4)
O22—Ti2—N21—C232	−36.8 (4)	C12—C119—C120—C121	95.5 (4)
O21—Ti2—N21—C232	75.7 (4)	C125—C120—C121—C122	−0.3 (6)
N22—Ti2—N21—C232	−161.1 (4)	C119—C120—C121—C122	−179.9 (4)
O22—Ti2—N22—C234	150.2 (5)	C120—C121—C122—C123	−0.3 (6)
O21—Ti2—N22—C234	36.8 (6)	C121—C122—C123—C124	0.7 (7)
N21—Ti2—N22—C234	−84.8 (6)	C122—C123—C124—C125	−0.4 (7)
O22—Ti2—N22—C235	−33.8 (4)	C123—C124—C125—C120	−0.3 (7)
O21—Ti2—N22—C235	−147.2 (4)	C121—C120—C125—C124	0.6 (6)
N21—Ti2—N22—C235	91.2 (4)	C119—C120—C125—C124	−179.8 (4)
C13—O13—C11—C12	−17.6 (4)	O12—C119—C126—C127	131.3 (4)
C13—O13—C11—C16	−142.5 (3)	C120—C119—C126—C127	−109.6 (4)
C13—O14—C12—C11	−8.2 (4)	C12—C119—C126—C127	14.5 (5)
C13—O14—C12—C119	−132.7 (3)	O12—C119—C126—C131	−49.1 (4)
O13—C11—C12—O14	15.4 (4)	C120—C119—C126—C131	70.0 (4)
C16—C11—C12—O14	134.7 (3)	C12—C119—C126—C131	−165.9 (3)
O13—C11—C12—C119	135.2 (3)	C131—C126—C127—C128	−1.3 (6)
C16—C11—C12—C119	−105.5 (4)	C119—C126—C127—C128	178.3 (4)
C11—O13—C13—O14	13.0 (5)	C126—C127—C128—C129	0.8 (7)
C11—O13—C13—C15	130.7 (5)	C127—C128—C129—C130	−0.5 (7)
C11—O13—C13—C14	−102.4 (4)	C128—C129—C130—C131	0.6 (7)
C12—O14—C13—O13	−2.3 (5)	C129—C130—C131—C126	−1.1 (6)
C12—O14—C13—C15	−120.1 (5)	C127—C126—C131—C130	1.5 (6)
C12—O14—C13—C14	112.9 (5)	C119—C126—C131—C130	−178.1 (4)
Ti1—O11—C16—C17	120.4 (4)	C28—C29—C210—C211	−1.1 (8)
Ti1—O11—C16—C113	−122.4 (5)	C29—C210—C211—C212	−0.2 (7)
Ti1—O11—C16—C11	−2.1 (6)	C210—C211—C212—C27	0.6 (7)
O13—C11—C16—O11	170.5 (3)	C28—C27—C212—C211	0.3 (6)
C12—C11—C16—O11	53.4 (4)	C26—C27—C212—C211	176.2 (4)
O13—C11—C16—C17	50.7 (4)	O21—C26—C213—C214	−18.9 (5)
C12—C11—C16—C17	−66.4 (4)	C27—C26—C213—C214	−135.5 (4)
O13—C11—C16—C113	−72.2 (4)	C21—C26—C213—C214	100.2 (4)
C12—C11—C16—C113	170.8 (3)	O21—C26—C213—C218	161.0 (4)
O11—C16—C17—C112	163.0 (4)	C27—C26—C213—C218	44.4 (5)
C113—C16—C17—C112	46.6 (5)	C21—C26—C213—C218	−79.9 (5)
C11—C16—C17—C112	−78.1 (5)	C218—C213—C214—C215	1.1 (6)
O11—C16—C17—C18	−15.3 (5)	C26—C213—C214—C215	−179.0 (4)
C113—C16—C17—C18	−131.7 (4)	C213—C214—C215—C216	−0.1 (7)
C11—C16—C17—C18	103.6 (4)	C214—C215—C216—C217	−1.2 (7)
C112—C17—C18—C19	−0.7 (6)	C215—C216—C217—C218	1.3 (8)
C16—C17—C18—C19	177.7 (4)	C214—C213—C218—C217	−0.9 (7)
C17—C18—C19—C110	0.5 (7)	C26—C213—C218—C217	179.1 (4)
C23—O23—C21—C22	−13.1 (4)	C216—C217—C218—C213	−0.2 (8)
C23—O23—C21—C26	−137.6 (4)	Ti2—O22—C219—C226	141.9 (3)
C23—O24—C22—C21	−11.8 (4)	Ti2—O22—C219—C220	−99.0 (4)
C23—O24—C22—C219	−137.1 (4)	Ti2—O22—C219—C22	20.1 (5)
O23—C21—C22—O24	14.7 (4)	O24—C22—C219—O22	174.3 (3)
C26—C21—C22—O24	134.5 (3)	C21—C22—C219—O22	56.5 (4)
O23—C21—C22—C219	134.7 (3)	O24—C22—C219—C226	54.3 (4)
C26—C21—C22—C219	−105.6 (4)	C21—C22—C219—C226	−63.5 (4)
C21—O23—C23—O24	6.4 (5)	O24—C22—C219—C220	−70.0 (4)
C21—O23—C23—C25	123.6 (6)	C21—C22—C219—C220	172.3 (3)
C21—O23—C23—C24	−111.2 (5)	O22—C219—C220—C225	130.9 (4)
C22—O24—C23—O23	4.1 (5)	C226—C219—C220—C225	−110.3 (4)
C22—O24—C23—C25	−112.5 (5)	C22—C219—C220—C225	15.2 (5)
C22—O24—C23—C24	121.4 (6)	O22—C219—C220—C221	−49.1 (4)
Ti2—O21—C26—C213	110.9 (4)	C226—C219—C220—C221	69.7 (4)
Ti2—O21—C26—C27	−131.7 (4)	C22—C219—C220—C221	−164.8 (3)
Ti2—O21—C26—C21	−11.8 (6)	C225—C220—C221—C222	−1.9 (6)
O23—C21—C26—O21	174.4 (3)	C219—C220—C221—C222	178.1 (4)
C22—C21—C26—O21	57.8 (4)	C220—C221—C222—C223	0.4 (7)
O23—C21—C26—C213	53.6 (4)	C221—C222—C223—C224	1.0 (7)
C22—C21—C26—C213	−63.1 (4)	C222—C223—C224—C225	−0.8 (8)
O23—C21—C26—C27	−68.8 (4)	C221—C220—C225—C224	2.0 (6)
C22—C21—C26—C27	174.6 (3)	C219—C220—C225—C224	−178.0 (4)
O21—C26—C27—C28	−39.7 (5)	C223—C224—C225—C220	−0.7 (8)
C213—C26—C27—C28	78.7 (4)	O22—C219—C226—C227	−20.6 (5)
C21—C26—C27—C28	−156.3 (4)	C220—C219—C226—C227	−137.5 (4)
O21—C26—C27—C212	144.5 (4)	C22—C219—C226—C227	97.5 (4)
C213—C26—C27—C212	−97.1 (4)	O22—C219—C226—C231	159.3 (4)
C21—C26—C27—C212	27.8 (5)	C220—C219—C226—C231	42.5 (5)
C212—C27—C28—C29	−1.7 (6)	C22—C219—C226—C231	−82.5 (5)
C26—C27—C28—C29	−177.8 (4)	C231—C226—C227—C228	−0.2 (6)
C27—C28—C29—C210	2.1 (8)	C219—C226—C227—C228	179.8 (4)
C18—C19—C110—C111	−0.1 (7)	C226—C227—C228—C229	1.3 (7)
C19—C110—C111—C112	−0.1 (7)	C227—C228—C229—C230	−1.0 (8)
C110—C111—C112—C17	−0.1 (7)	C228—C229—C230—C231	−0.3 (8)
C18—C17—C112—C111	0.5 (6)	C227—C226—C231—C230	−1.2 (7)
C16—C17—C112—C111	−177.8 (4)	C219—C226—C231—C230	178.9 (4)
O11—C16—C113—C118	139.3 (4)	C229—C230—C231—C226	1.4 (7)
